# Factors Influencing Postoperative Inpatient Rehabilitation Requirement After Surgical Intervention for Isolated Hip Fracture: A Multicenter Study

**DOI:** 10.1111/os.14290

**Published:** 2024-11-15

**Authors:** Daniel J. Lynch, Andrew Romero, James P. McFadden, Peter Zeblisky, Huazhi Liu, Darwin Ang

**Affiliations:** ^1^ Department of Orthopaedic Surgery Ocala Florida USA; ^2^ University of Central Florida College of Medicine Orlando Florida USA

**Keywords:** arthroplasty, hip fracture, orthopedics, rehabilitation, trauma

## Abstract

**Purpose:**

Hip fractures in the elderly, especially those discharged to a rehab facility, have historically been associated with poor outcomes. There has yet to be identified which patients have a higher likelihood of a rehab discharge after isolated hip fracture fixation. The purpose of this study was to identify factors that predispose patients to require short or long‐term rehab after surgical intervention of traumatic, isolated hip fractures.

**Methods:**

Patients undergoing operative management of hip fractures (*n* = 71,849) from 2017 to 2019 at institutions that submitted data to a nationwide database were analyzed retrospectively. Various factors were compared between patients discharged to inpatient rehab (*n* = 56,178) versus home (*n* = 15,671).

**Results:**

The rehab discharge group was significantly older and predominantly female. This cohort had a longer average hospital stay, higher incidence of diabetes, congestive heart failure, chronic renal failure, history of cerebrovascular accident, functionally dependent health status, hypertension, chronic obstructive pulmonary disease, dementia, baseline anticoagulation therapy, and history of myocardial infarction. DVT during hospitalization was encountered more often in patients discharged to rehab. Patients with femoral neck fractures and those undergoing total hip arthroplasty were more often discharged home. Patients with intertrochanteric hip fractures and those undergoing fracture fixation were more often discharged to rehab.

**Conclusions:**

Multiple risk factors associated with a significantly higher likelihood of a rehab discharge after isolated hip fracture surgery were identified. Early identification of these patients may provide an opportunity to optimize patients for home discharge and better outcomes.

**Level of Evidence:**

Level III, Case‐Control Study.

## Graphical abstract

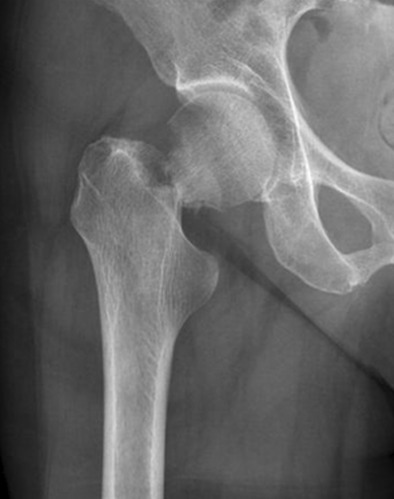

## 1 Introduction

Hip fractures have traditionally been associated with negative outcomes in the orthopedic literature [1]. As we expect the incidence of hip fractures to exceed 6 million across the world per year in the next 25 years, these injuries and their ramifications are something to pay close attention to [1, 2, 3, 4]. When it comes to hip fractures, extra‐capsular location, delay in surgical fixation, increasing age, reduced cognitive ability, and the presence of co‐morbidities are known negative predictors after operative intervention for hip fractures [3]. An important prognostic outcome factor after surgery is discharge destination. An individual's hospital course can influence one's recovery and overall well‐being after hip fracture surgery, for better or worse. It has been postulated that an interdisciplinary, patient‐centered approach may benefit this elderly hip fracture population. Bano et al. demonstrated that compared to a traditional hospital care model, an orthogeriatric interdisciplinary approach to hospital care after an elderly hip fracture led to a higher incidence of living at home 6 months after surgery, less functional decline, higher activities of daily living (ADL) scores, and a higher probability of independent walking ability [5].

Previous research shows that discharge to a skilled nursing (SNF) or rehab facility is associated with inferior outcomes, higher costs, and a higher incidence of infection [6, 7, 8, 9, 10]. For example, in their study looking at discharge destination and post‐discharge outcomes after revision total hip or total knee arthroplasty, Keswani et al. showed that those patients discharged to either SNF or an acute inpatient rehab facility, compared to those discharged home, saw a significantly higher rate of 30‐day severe adverse events and unplanned readmissions [9]. After elective total knee arthroplasty, Jorgenson et al. demonstrated that patients discharged to either acute inpatient rehab facilities or SNFs had odds ratios of 7.76 and 2.01, respectively, of 30‐day readmission compared to those discharged home [10]. Investigative studies focusing on hip arthroplasty patient outcomes, with most studies looking at elective joint replacement surgery, have reported that older patients residing alone and those who are non‐independent ambulators are more likely to be discharged to a SNF or rehab facility [11, 12, 13, 14]. One's inpatient postoperative courses can influence discharge destination. An important prognostic event is the initial postoperative evaluation by physical therapy (PT). Increasing age, longer hospital stay, poor pre‐injury baseline functional status, and extra capsular hip fracture location have all been found to be associated with “worse” initial PT evaluation scores. Patients with these characteristics thus have a higher likelihood of ending up at a SNF or rehab facility after hip fracture surgery [15].

It has been postulated that an interdisciplinary patient‐centered approach may benefit this elderly hip fracture population. Bano et al. demonstrated that compared to a traditional hospital care model, an orthogeriatric interdisciplinary approach to hospital care after an elderly hip fracture led to a higher incidence of living at home 6 months after surgery, less functional decline, higher activities of daily living (ADL) scores, and a higher probability of independent walking ability [5]. Factors that increase patients' likelihood of being discharged to rehab over home after surgical intervention of traumatic isolated hip fractures have not been investigated on a large scale. The purpose of this study was as follows: (1) identify patient demographic, comorbidity, injury, and hospital stay factors associated with a higher likelihood of a postoperative rehab discharge destination; (2) identify any fixation technique of isolated hip fractures that increases patients' likelihood of a postoperative rehab discharge destination; (3) summarize patient and fixation method characteristics that increase the risk of a rehab discharge destination after isolated hip fracture surgery.

## 2 Methods

### 2.1 Study Design and Population

This is a retrospective case‐control study of all level 1 and 2 trauma centers in the United States verified by the American College of Surgeons (ACS), which was exempt from obtaining IRB. The index population was patients aged 18–89 years who underwent surgical intervention for isolated hip fractures from 2017 to 2019 at institutions participating in the ACS Trauma Quality Improvement Program (TQIP) database (*n* = 71,849). For patient selection, ICD‐10 codes for both displaced and non‐displaced femoral neck, intertrochanteric, and sub trochanteric fractures were used to generate our isolated hip fracture cohort (ages 18–89). Procedure codes were then used to stratify patients into three groups based on the fixation method: open reduction internal fixation (ORIF), hemiarthroplasty, or total hip arthroplasty.

### 2.2 Cohorts and Outcomes

Outcomes were divided into those discharged to rehab centers versus those discharged home after surgery. Various demographic, comorbidity, injury, and hospital stay variables were analyzed between the two groups; rehab (*n* = 56,178) versus home (*n* = 15,671) (Table 1). Cohorts were then stratified by surgery type: ORIF, hemiarthroplasty, and total hip arthroplasty. The cohorts were also stratified by post‐surgical complications, including deep surgical site infection, deep venous thrombosis (DVT), and superficial surgical site infection (Table 2).

**TABLE 1:** Rehab versus home discharge group comparison.

	Rehab *N* = 56,178	Home *N* = 15,671	*p* value
**Demographics**			
Age (years)	76.6 (±9.9)	65.4 (±14.9)	< *0.0001*
Gender			
Male	32.9%	41.5%	< *0.0001*
Female	67.1%	58.5%	
Race			
Caucasian	90.2%	87.8%	< *0.0001*
African‐American	4.9%	5.5%	*0.003*
Asian	1.4%	2.0%	< *0.0001*
Other	3.5%	4.8%	< *0.0001*
Advanced directive limiting care	7.9%	5.8%	< *0.0001*
Functionally dependent health status	23.6%	16.3%	< *0.0001*
***Comorbidities***			
Smoker	13.1%	24.5%	< *0.0001*
Diabetes	26.3%	17.7%	< *0.0001*
Alcohol use disorder	3.1%	4.8%	< *0.0001*
Currently receiving chemotherapy for cancer	1.02%	0.97%	0.55
Congenital anomalies	0.5%	0.6%	*0.04*
Congestive heart failure	9.5%	4.7%	< *0.0001*
Chronic renal failure	4.2%	1.9%	< *0.0001*
History of cerebrovascular accident	6.6%	4.0%	< *0.0001*
Disseminated cancer	1.2%	1.0%	0.10
Hypertension	66.2%	47.8%	< *0.0001*
Prematurity	0.02%	0.03%	0.63
Chronic obstructive pulmonary disease	16.4%	12.1%	< *0.0001*
Steroid use	2.2%	1.7%	*0.001*
Cirrhosis	1.2%	1.3%	0.42
Dementia	16.1%	10.3%	< *0.0001*
Anticoagulant therapy	17.5%	9.5%	< *0.0001*
Angina pectoris	0.3%	0.2%	*0.01*
Mental/personality disorder	13.0%	12.5%	0.09
Myocardial infarction	1.6%	0.9%	< *0.0001*
Peripheral arterial disease	2.1%	1.3%	< *0.0001*
Drug abuse disease	2.2%	4.5%	< *0.0001*
***Injury factors***			
*Fracture location*			
Femoral neck fracture	24.5%	42.5%	< *0.0001*
Intertrochanteric	63.5%	46.2%	< *0.0001*
Sub trochanteric	5.4%	5.4%	0.82
Work‐related (Y/N)	1.1%	4.4%	< *0.0001*
Transport mode			
Ground ambulance	91.1%	79.1%	< *0.0001*
Helicopter ambulance	0.6%	1.1%	< *0.0001*
Fixed‐wing ambulance	0.2%	0.6%	< *0.0001*
Private/public vehicle/walk‐in	7.8%	18.9%	< *0.0001*
Trauma center criteria: Glasgow coma scale < 13	0.2%	0.1%	0.10
Trauma center criteria: systolic blood pressure < 90 mmHg	0.11%	0.06%	0.10
Pre‐hospital cardiac arrest	0.19%	0.22%	0.42
***Hospital stay factors***			
Total intensive care unit length of stay	3.9 (±3.5)	3.7 (±3.9)	0.35
Time from presentation to final discharge	6.6 (±16.1)	5.6 (±4.2)	< *0.0001*
Time to: DVT prophylaxis	2.3 (±1.3)	2.3 (±1.2)	0.19
Facility level: hospital type			
For profit	14.0%	11.6%	< *0.0001*
Non‐profit	85.6%	87.7%	< *0.0001*
Government	0.4%	0.7%	< *0.0001*
Blood transfusion Y/N	0.2%	0.1%	0.08
Blood transfusion measurement (mL)	438 (±296)	373 (±194)	0.34

*Note*: Italicized values indicate statistical significance, *p*‐value set at 0.05.

**TABLE 2:** Multivariate regression analysis adjusted by age, race, comorbidities, dependent health status, anticoagulation therapy, alcohol/drug abuse.

	Rehab *N* = 56,178	Home *N* = 15,671	Adjusted *p*‐value *	Adjusted OR * (Reference=‘Rehab’)
***Type of procedure***				
Open reduction and internal fixation	90.1%	83.5%	< *0.0001*	*0.57* (*0.54*, *0.60*)
Hemiarthroplasty	0.8%	0.8%	0.43	1.09 (0.88, 1.36)
Total hip arthroplasty	9.5%	15.9%	< *0.0001*	*1.75* (*1.65*, *1.86*)
***Complications***				
Deep surgical site infection	0.02%	0.01%	0.09	0.16 (0.02, 1.36)
Deep venous thrombosis	0.33%	0.19%	*0.004*	*0.54* (*0.35*, *0.83*)
Superficial surgical site infection	0.02%	0.01%	0.13	0.19 (0.02, 1.66)

*Note*: Italicized values indicate statistical significance, *p*‐value set at 0.05.

### 2.3 Statistical Analysis

All data were analyzed by a local statistician associated with our trauma department using SAS version 9.4 (SAS Institute Inc., Cary, NC). Descriptive variables were compared using chi‐square tests for categorical variables and t‐tests for continuous variables. A multivariable regression analysis, controlling for age, race, comorbidities, dependent health status, anticoagulation therapy, and substance abuse, was used to compare methods of surgical intervention as well as post‐surgical complications between both groups.

## 3 Results

**FIGURE 1:**
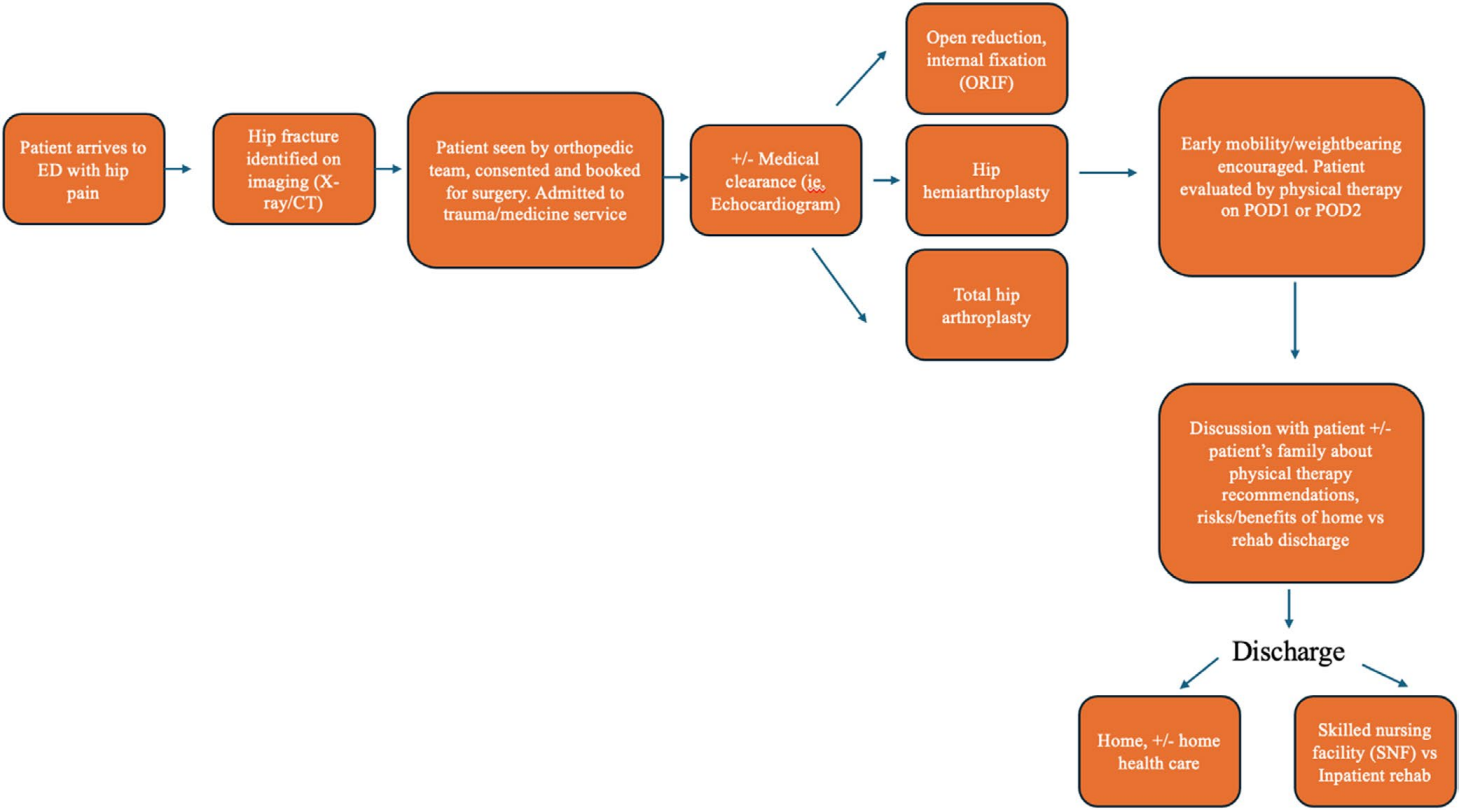
Hip fracture hospital course flowchart.

Figure 1 is a flowchart depicting the usual hospital course for hip fracture patients, from when they arrive at the emergency department to when they are discharged. At our institution, skeletal or skin traction is not typically used for either intra‐ or extra‐capsular hip fractures. After patients are triaged, they are admitted overnight for pain control until surgery the next day, pending any medical clearance. Although we cannot determine how patients not from our institution are managed before fixation, there is evidence discouraging the use of skin and skeletal traction for proximal femur fractures before definitive fixation [16]. Table 1 compares demographic, comorbidity, injury, and hospital stay variables between patients postoperatively discharged to rehab (*n* = 56,178) and patients discharged home (*n* = 15,671).

### 3.1 Demographics

Patients postoperatively discharged to rehab were significantly older, more often Caucasian and female. They also significantly more often had an advanced directive limiting care and a “functionally dependent” health status.

### 3.2 Comorbidities

Patients postoperatively discharged to rehab were had a significantly larger proportion of patients with the following comorbidities: diabetes, congestive heart failure, chronic renal failure, hypertension (HTN), chronic obstructive pulmonary disease (COPD), dementia, angina pectoris, peripheral arterial disease (PAD), history of steroid use, cerebrovascular accident (CVA), myocardial infarction (MI), and anticoagulation therapy use. It also had a significantly smaller proportion of smokers, those with alcohol use disorder, and those with drug substance abuse disorders.

### 3.3 Injury Factors

Patients postoperatively discharged to rehab were less likely to have a work‐related injury. It also showed a substantially larger proportion of patients who arrived via private or public vehicle than helicopter and ambulance. Regarding fracture pattern, this cohort had a significantly smaller proportion of patients with femoral neck fractures and a considerably larger proportion of patients with intertrochanteric hip fractures.

#### 3.3.1 Hospital Stay Factors

Patients postoperatively discharged to rehab experienced a significantly longer average hospital stay. It also had a considerably larger proportion of patients staying at a for‐profit hospital. No significant differences were found between the cohorts regarding DVT prophylaxis or the need for a blood transfusion.

### 3.4 Multivariate Regression Analysis

Table 2 shows the results of our regression analysis when controlling for age, race, comorbidities, dependent health status, anticoagulation therapy, and drug or alcohol abuse. Patients who underwent ORIF had significantly lower odds of being discharged home. Patients who underwent total hip arthroplasty (THA) had significantly higher odds of being discharged home. No significant difference in discharge destination was found amongst patients who underwent hip hemiarthroplasty. Patients discharged home had significantly lower odds of experiencing a postoperative DVT.

## 4 Discussion

### 4.1 Predictors of Rehab Discharge

Older age, multiple medical comorbidities, low preoperative functional status, and a longer hospital admission were all significant predictors of eventual discharge to SNF or rehab. Interestingly, those undergoing ORIF were significantly more often facing a post‐operative discharge to rehab. Those who underwent THA were discharged home significantly more often than those undergoing ORIF. A study looking at total joint arthroplasty in the knee and hip showed that one's discharge destination expectation surrounding their hospital stay is a strong prognosticator of discharging to SNF [13]. In our patient cohort, those undergoing THA were likely somewhat influenced by a preoperative discussion with the surgeon on the evidence showing any of the well‐known negative sequela related to a SNF or rehab discharge after operative hip fracture treatment [6, 7, 8, 9, 10, 17].

In the elderly, hip ORIF on the other hand, has been shown to be an independent prognosticator of a SNF or rehab discharge after hip fracture surgery [18]. One study showed that their ORIF cohort, compared to patients undergoing hip arthroplasty, demonstrated decreased weight bearing on the operative limb in the 21 days after surgery [19]. In the present study, this phenomenon may have affected patients' postoperative physical therapy evaluation and/or delayed full mobilization after surgery, thus leading to a higher probability of a postoperative SNF or rehab destination [14]. It is also worth highlighting how socioeconomic status may influence postoperative discharge destination. A study by Kristensen et al. demonstrated that higher education level and family income both significantly reduced re‐admission rate and 30‐day mortality after hip fracture surgery [20]. One's level of education and family income, which is likely related to the availability of assistance at home after hip fracture surgery, although not analyzed in this study, likely contributes to patients' ability to be discharged home after surgery confidently.

### 4.2 Patient Age

Although our study included patients aged 18–89, most of the patients in our cohort were between the ages of 65 and 75. However, it is worth briefly mentioning how age may play a role in outcomes after sustaining a hip fracture. In their study looking at isolated hip fractures in patients < 60 years old, Coughlin et al. demonstrated that even in these “younger” patients, there was a significant reduction in Oxford Hip Scores, ADL scores, and visual analog scale scores at a mean of 57‐month follow‐up [21]. In their cohort of “older adults” who sustained proximal femur fractures, Ceolin et al. suggest that patients experience the greatest functional loss, as measured by ADL scores, in the first 6 months postoperatively, which can increase the risk of 1‐year mortality [22]. These two studies highlight that even in “younger” patients, full recovery of function and independence after a hip fracture may take years. In “older” patients, this full recovery may never occur. Although a small piece of the puzzle, immediate postoperative discharge destination is usually the patient's first step towards regaining functionality and independence.

### 4.3 Clinical Utility

Suppose patients sustaining traumatic isolated hip fractures with the identified risk factors can be more promptly identified upon hospital arrival. In that case, there may be an opportunity for a streamlined optimization and education process to increase their chances for an improved postoperative course and outcome. Based on previous data on a home discharge after hip surgery compared to rehab discharge, this could help reduce re‐admission and re‐operation rates and overall costs [23, 24].

#### 4.3.1 Postoperative DVT

Especially in the elderly, postoperative DVT has been linked to an increased length of stay after hip fracture surgery [25]. There are numerous studies demonstrating the incidence of this potentially life‐threatening event, DVT, after hip fracture fixation [26, 27, 28, 29, 30, 31]. When controlling for age, race, comorbidities, dependent health status, history of anticoagulation therapy, and substance use, we demonstrated that amongst our hip fracture cohort, patients who experienced a DVT during their hospital stay had significantly higher odds of discharging to SNF or rehab. Prolonged immobilization after surgery, which likely contributed to the incidence of DVT observed, may also have contributed to the deconditioning of patients and therefore led to a higher perceived need for rehab from both the patient and the inpatient physical therapy provider. Emphasis on appropriate medical workup for increased DVT risk, early postoperative mobilization, and proper anticoagulation therapy could improve outcomes and increase rates of postoperative home discharge.

### 4.4 Limitations and Strengths

Our goal was to analyze factors influencing a postoperative SNF or rehab destination after surgical intervention for isolated hip fractures. Our large cohort included over 71,000 patients from a database spanning multiple level I and II trauma centers across the country, increasing the generalizability of our results. In addition, this cohort, consisting of traumatic isolated hip fractures requiring hospitalization and surgical intervention, has not been as extensively studied in the past regarding postoperative outcomes and discharge destinations as it has been for elective total joint replacements. As stated previously, the majority of studies analyzing the outcomes related to discharge destination have been after elective total joint replacement.

Limitations of our study include the retrospective nature of our data collection, which precludes a direct cause and effect but only suggests a strong association. Patients from this study were identified using ICD codes, which depend on correct coding by billers and providers and increasing the risk of misclassification bias. Specific reasons why patients were discharged to SNF or rehab instead of home could not be identified, and there was no postoperative follow‐up data available for analysis. We attempted to capture and analyze relevant “factors” related to hip fractures and being in the hospital for our study. Still, we understand that many more variables, from the time of injury to the time of discharge, play a role in one's potential postoperative destination. We also understand that not every patient can “feasibly” have the option to be discharged home despite being able to ambulate postoperatively. In addition to the aforementioned socioeconomic factors of education level and family income, circumstances such as the presence of stairs at the residence or the presence of assistance or lack thereof likely have a significant impact on the perceived ability to go home postoperatively irrespective of postoperative performance with physical therapy. This also may differ across countries where there is likely a significant difference in the availability of home health services, SNFs, and rehab centers. This must be kept in mind with the results of our study.

### 4.5 Future Study

Future studies of this patient population could aim to identify organized ways to optimize patients with the risk factors identified in this study for a home discharge after surgery. Along with the hip fracture protocol at our institution, which includes a goal of hip fracture surgical intervention within 24 h of patient arrival, there may be utility with perhaps earlier physical therapy evaluation and intervention, prompt medicine specialist consultation and collaboration, and surgeon‐patient discussion regarding home versus rehab discharge. Appropriate long‐term follow‐up of that patient cohort could determine the success of these implementations.

## 5 Conclusion

There is a surplus of data showing the deleterious outcomes of a SNF or rehab destination after elective total joint surgery, and this study creates a framework for identifying at‐risk patients in the acute trauma setting for isolated hip fractures. As previously stated, the well‐known increased morbidity and mortality accompanying hip fractures in the elderly, along with the findings in this study, presents a good opportunity for orthopedic providers to identify and intervene in these at‐risk patients [32, 33]. With hip fractures, these patients lose mobility and become candidates for infection, pressure sores, pneumonia, urinary tract infections, and blood clots [25, 34, 35]. If patients are postoperatively discharged to SNF or rehab, the data shows that these occurrences are even more likely. Regardless of whether patients are undergoing ORIF, hemi or total hip arthroplasty for isolated hip fractures in the acute trauma setting, there exists an opportunity for early identification and intervention for patients who, based on the risk factors we identified, are more likely to discharge to SNF or rehab postoperatively. These identifiable risk factors may give us a chance to address the known increase in morbidity and mortality risk profiles that are associated with discharge to rehab following surgical intervention of hip fractures.

## Author Contributions

**Daniel J. Lynch:** conceptualization, methodology, validation, investigation, writing – original draft, writing – review and editing. **Andrew Romero:** conceptualization, validation, investigation, resources, writing – original draft, writing – review and editing. **James P. McFadden:** supervision, writing – review and editing, validation. **Peter Zeblisky:** writing – review and editing, resources, methodology, conceptualization. **Huazhi Liu:** formal analysis, methodology, supervision, data curation, investigation. **Darwin Ang:** writing – review and editing, supervision, project administration, validation, conceptualization.

## Conflicts of Interest

The authors declare no conflicts of interest.
